# Spectroflourometric and Spectrophotometric Methods for the Determination of Sitagliptin in Binary Mixture with Metformin and Ternary Mixture with Metformin and Sitagliptin Alkaline Degradation Product

**Published:** 2011-03

**Authors:** Ramzia I. El-Bagary, Ehab F. Elkady, Bassam M. Ayoub

**Affiliations:** *Department of Pharmaceutical Chemistry, Faculty of Pharmacy, Cairo University, Kasr El-Aini St., Cairo, Egypt*

**Keywords:** metformin, sitagliptin phosphate, spectrophotometry, stability indicating assay, flourometry, pharmaceutical preparation

## Abstract

Simple, accurate and precise spectroflourometric and spectrophotometric methods have been developed and validated for the determination of sitagliptin phosphate monohydrate (STG) and metformin HCL (MET). Zero order, first derivative, ratio derivative spectrophotometric methods and flourometric methods have been developed. The zero order spectrophotometric method was used for the determination of STG in the range of 50-300 μg mL^-1^. The first derivative spectrophotometric method was used for the determination of MET in the range of 2–12 μg mL^-1^ and STG in the range of 50-300 μg mL^-1^ by measuring the peak amplitude at 246.5 nm and 275 nm, respectively. The first derivative of ratio spectra spectrophotometric method used the peak amplitudes at 232 nm and 239 nm for the determination of MET in the range of 2–12 μg mL^-1^. The flourometric method was used for the determination of STG in the range of 0.25-110 μg mL^-1^. The proposed methods used to determine each drug in binary mixture with metformin and ternary mixture with metformin and sitagliptin alkaline degradation product that is obtained after alkaline hydrolysis of sitagliptin. The results were statistically compared using one-way analysis of variance (ANOVA). The methods developed were satisfactorily applied to the analysis of the pharmaceutical formulations and proved to be specific and accurate for the quality control of the cited drugs in pharmaceutical dosage forms.

## INTRODUCTION

**Figure 1:**
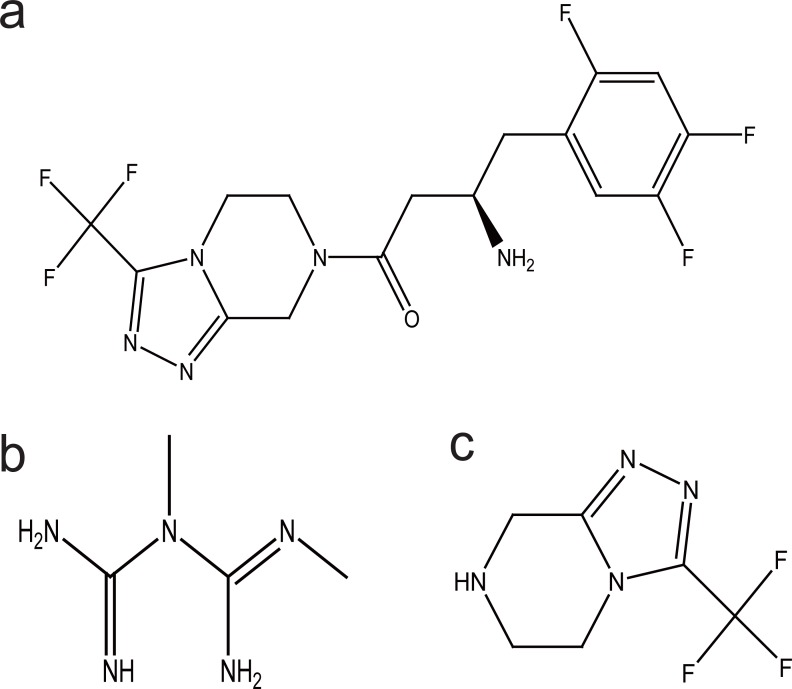
Chemical structures of sitagliptin (a), metformin (b) and sitagliptin alkaline degradation product (c).

Sitagliptin (STG), [(2*R*)-1-(2,4,5-trifluorophenyl)-4-oxo-4-[3-(trifluoromethyl)- 5,6 dihydro [1,2,4] triazolo [4,3-*α*]pyrazin-7(8*H*)-yl] butan-2-amine] (Fig. 1a) and meformin (MET), N,N-dimethylimidodicarbonimidic diamide (Fig. 1b) are two well known hypoglycemic drugs. STG is a novel oral hypoglycemic drug of the dipeptidyl peptidase 4 inhibitor class (1). DPP-4 inhibitors represent a new therapeutic approach to the treatment of type 2 diabetes that functions to stimulate glucose-dependent insulin release and reduce glucagons levels. This is done through inhibition of the inactivation of incretins, particularly glucagon-like peptide-1 (GLP-1) and gastric inhibitory polypeptide (GIP), thereby improving glycemic control (2-4). STG is used as a single therapy or in combination with MET. MET is a biguanide drug effective in patients who lack functioning islet cells as it act by simulations of glycolysis in peripheral tissues (7, 8).

Literature survey reveals that liquid chromatographic methods have been developed for the determination of STG in biological fluids (2, 5, 6). It is worth noting that only one method has been adopted for the determination of STG in its pharmaceutical formulation. This method was based on colorimetric determination of STG after its reaction with formaldehyde and acetylacetone (1). Besides, several methods have been reported for determination of MET in pharmaceutical preparations and biological fluids including LC/MS/MS (7) and HPLC (8-11).

Spectrofluorometry has long been applied in the field of pharmaceutical analysis of many drugs (12-15) because of the higher sensitivity than is attainable in absorption spectrophotometry. A necessary condition for a compound to fluoresce is that it absorbs light in the UV or visible region of the spectrum. Accordingly, compounds that have a conjugated π- electron system may give efficient re-emission of the absorbed energy as a direct method for the determination in which the native fluorescence of the molecule is measured (12-15). On the other hand, spectrophotometry continues to be very popular, because of its simplicity and low cost so it has long been applied for the analysis of many drugs (15-21). This study presents the determination of STG and MET in binary mixture and in ternary mixture with STG degradation product, 3-(trifluoromethyl)-5,6,7,8-tetrahydro-[1,2,4]triazolo[4,3-a]pyrazine (Fig. 1c) which was prepared by degradation of STG using alkaline hydrolysis and its structure was elucidated by different spectroscopic techniques to ensure the product. It is also reported that it is the synthetic intermediate of STG (22) and it is the active metabolite of the drug (23).

## EXPERIMENTAL

### Instrumentation

A Jenway 6800 double beam ultraviolet/visible spectrometer, U.K., connected to an IBM compatible computer with 1-cm quartz cell and supported with Jenway flight deck software was used. BIO-TEK spectrofluorometer, Italy, SFM 25 was used.

### Reagents and reference samples

Pharmaceutical grade sitagliptin phosphate monohydrate, certified to contain 99.80%, Januvia^®^ tablets nominally containing 128.5 mg of sitagliptin phosphate monohydrate per tablet (batch no. S0273) and Janumet^®^ tablets nominally containing 64.25 mg of sitagliptin phosphate monohydrate and 1000 mg of metformin per tablet (batch no. 0426570) were kindly supplied from Merck Sharp and Dohme Co. (Cairo, Egypt). pharmaceutical grade metformin hydrochloride, certified to contain 99.79% was kindly supplied by Chemical Industries Development (Cid) Co. (Giza, Egypt). Standard stock solutions of each drug (1 mg/ml) were prepared by dissolving 100 mg of the drug in methanol and completing the volume to 100 ml in a volumetric flask and then the required concentrations were prepared by serial dilution. All the solvents used were of analytical grade.

**Preparation of alkaline degradation product.** An amount of 1 g of STG bulk powder was dissolved in 250 ml of 5N aqueous sodium hydroxide and the solution was refluxed for 6 hours on a boiling water bath, cooled and neutralized by 5N aqueous hydrochloric acid. The formed precipitate was filtered, washed several times and dried. Complete degradation was confirmed using TLC plates and its structure was elucidated by different spectroscopic techniques.

### General procedures and calibration graphs

**Flourometric method.** Aliquots from STG stock standard solution equivalent to 2.5-1100 μg were accurately measured and transferred into a set of 10 mL volumetric flasks and the volumes were completed with methanol. The relative fluorescence intensity was measured at the specified excitation and emission wavelengths (λem at 575 nm with λex at 263 nm), then plotted against its corresponding concentration and the regression parameters were computed.

**Zero order spectrophotometric method.** Aliquots from STG stock standard solution equivalent to 0.5-3 mg were accurately measured and transferred into sets of 10 mL volumetric flasks and the volumes were completed with methanol. The zero order absorption spectra of each solution was recorded against methanol as a blank at 267 nm, then plotted against its corresponding concentration and the regression parameters were computed.

**First derivative spectrophotometric method.** Aliquots from MET and STG stock standard solutions equivalent to 20-120 μg and 0.5-3 mg, respectively, were accurately measured and transferred into two separate sets of 10 mL volumetric flasks and the volumes were completed with methanol. The zero order absorption spectra of each solution was recorded against methanol as a blank, then the first derivative spectra were computed applying scaling factor of 10. The amplitudes at 246.5 nm and 275 nm were measured for MET and STG, respectively, then plotted against corresponding concentrations and the regression parameters were computed.

**Derivative ratio spectrophotometric method.** The previously scanned zero order absorption spectra of MET were divided by the spectrum of STG (50 μg mL^-1^) which is the chosen divisor. The first derivative of the obtained ratio spectra was computed. Two calibration curves were constructed relating the amplitudes at 232 nm and 239 nm to the corresponding concentrations of MET.

**Assay of laboratory-prepared mixtures.** The absorption spectrum was recorded for the laboratory prepared mixtures, against methanol as a blank. The zero order absorption spectra at 267 nm was used for the direct determination of STG. The amplitudes of the first derivative spectra of the laboratory prepared mixtures containing different ratios of MET and STG were measured at 246.5 nm and 275 nm for MET and STG, respectively. The concentrations of MET and STG were calculated from their corresponding regression equations. The previously scanned zero order absorption spectra for the laboratory prepared mixture were divided by the spectrum of STG (50 μg mL^-1^).

For the spectroflourometric part, the relative fluorescence intensity of STG in the laboratory prepared mixtures was measured at the specified excitation and emission wavelengths (λem at 575 nm with λex at 263 nm).

**Assay of Januvia^®^ and Janumet^®^ tablets.** Twenty tablets of each drug were weighed and the coats were removed by carefully rubbing with a clean tissue wetted with methanol. An accurately weighed amount of the finely powdered Januvia^®^ and Janumet^®^ tablets equivalent to 100 mg of STG were separately made up to 100 ml with the selected solvent, the solution was filtered followed by serial dilution to the required concentration for each experiment. The procedure was continued as mentioned under general procedures and calibration.

## RESULTS AND DISCUSSION

Literature survey reveals that only liquid chromatographic methods have been developed for the determination of STG in biological fluids (2, 5, 6). The development of spectrophotometric and spectrofluorometric methods for the determination of STG either in binary mixture with MET or ternary mixture with MET and STG alkaline degradation product was of interest as no such methods have been reported for these mixtures.

STG alkaline degradation product (Fig. 1c) has been prepared using alkaline hydrolysis of the amide bond of the intact drug. Complete degradation was confirmed using TLC plates. Structure elucidation of the obtained secondary amine was confirmed using different spectroscopic techniques. I.R. spectrum showed the absence of the characteristic peak of the carbonyl group at 1639 cm^-1^. UV spectroscopy showed that the absence of the characteristic maximum of intact STG at 267 nm. Then Mass spectroscopy confirmed the hydrolyzed product showing the molecular weight of the obtained structure at 192.

**Figure 2:**
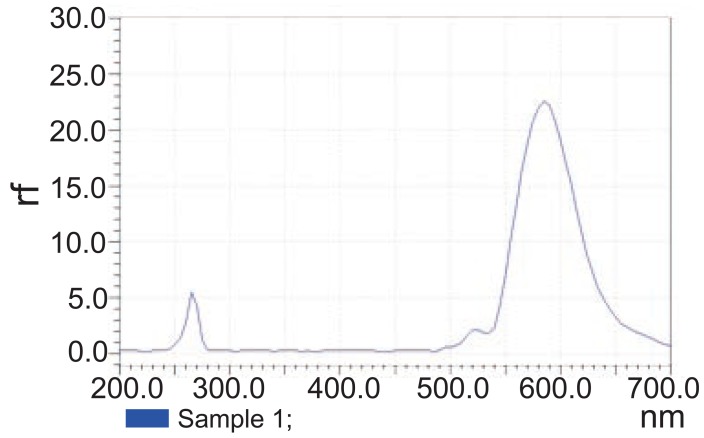
Excitation and emission spectra of sitagliptin (55 μg ml^-1^).

**Figure 3:**
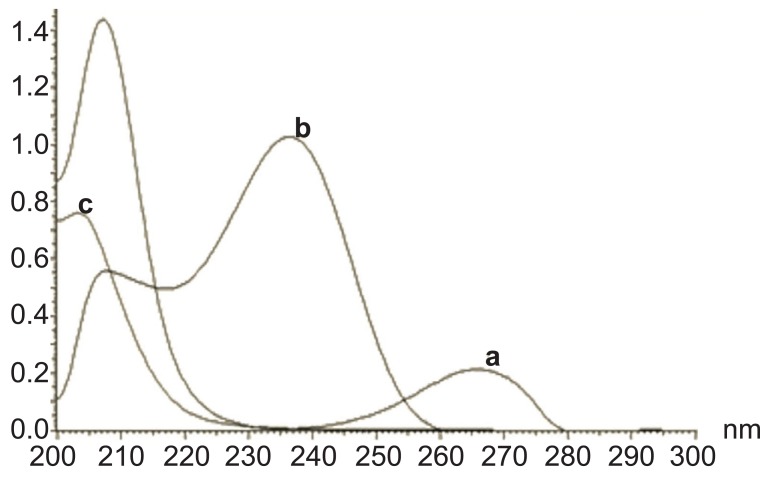
Zero order spectra of sitagliptin 50 μg ml^-1^ (a), metformin 10 μg ml^-1^ (b) and sitagliptin degradation product 50 μg ml^-1^ (c).

STG could be determined using flourometry (Fig. 2) and zero order spectrophotometry (Fig. 3) without interference from MET or STG alkaline degradation product. The characteristic parameters of the regression equations are given in Table 1 and Table 2. MET could not be determined by the previously mentioned methods as its absorption spectrum exhibits overlap from that of STG (Fig. 3) and it does not have any native fluorescence. First derivative, ratio derivative spectrophotometric methods have been applied to allow the resolution of the two drugs. MET was successfully determined in the presence of STG and STG degradation product.

**Table 1:** Results obtained by the proposed flourometric method for the determination of sitagliptin

λ_max_ excitation of measurements	263 nm
λ_max_ emission of measurements	575 nm
Linearity	0.25-110 μg ml^-1^
Regression equation	C_μg/ml_ = 0.4201 F*_575_ – 0.0065
Regression coefficient (r^2^)	1.00
LOD μg ml^-1^	0.05
LOQ μg ml^-1^	0.15
S_b_	5 × 10^-5^
S_a_	2.7 × 10^-3^
Confidence limit of the slope	0.4201 ± 6.23 × 10^-3^
Confidence limit of the intercept	-0.0065 ± 1.15 × 10^-4^
Standard error of the estimation	0.0062
**Results**	
Drug in bulk	99.83 ± 0.83
Drug in laboratory prepared binary mixture with MET	99.93 ± 0.70
Drug in laboratory prepared binary mixture with STG degradation product**	99.81± 0.60
Drug in laboratory prepared ternary mixture with MET and STG degradation product	99.94 ± 1.09
Drug in dosage form (Januvia^®^)	99.85 ± 0.98
Drug added	100.01 ± 0.64
Drug in combination with MET dosage form (Janumet^®^)	99.82 ± 0.54
Drug added	99.64 ± 0.74

* F, Relative flourescence;

** STG degradation product with different ratios (10% to 30%) w/w.

**Table 2:** Results obtained by zero order method for the determination of sitagliptin

λ_max_ of measurements	267 nm
Obedience of Beer’s law	50-300 μg ml^-1^
Regression equation	C_μg/ml_ = 0.0042 A_267_ + 0.0029
Regression coefficient (r^2^)	0.9999
LOD μg ml^-1^	2.88
LOQ μg ml^-1^	8.72
S_b_	4.54 × 10^-5^
S_a_	8.8 × 10^-3^
Confidence limit of the slope	0.0042 ± 2.03 × 10^-2^
Confidence limit of the intercept	0.0029 ± 1.05 × 10^-4^
Standard error of the estimation	0.0095
**Results**	
Drug in bulk	100.13 ± 0.67
Drug in laboratory prepared ternary mixture with MET and STG degradation product^a^	100.47 ± 1.09
Drug in dosage form (januvia^®^)	100.17 ± 0.81
Drug added	100.39 ± 0.83
Drug in combination with MET dosage form (janumet^®^)	100 ± 1.12
Drug added to the mixture	100.25 ± 1.43

a STG degradation product with different ratios (10% to 30%) w/w.

**Figure 4:**
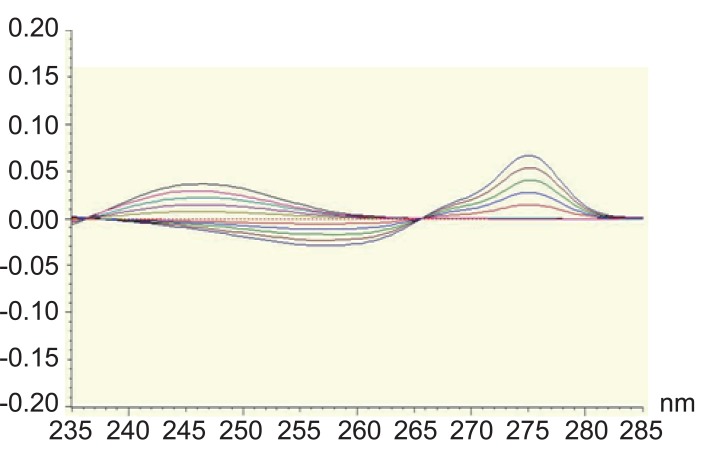
First order spectra of sitagliptin (50 to 250) μg ml^-1^ at 275 nm and metformin (2 to 10) μg ml^-1^ at 246.5 nm.

Direct UV-absorbance measurement is subject to interference from excepients and degradation products. Among the techniques used to eliminate such interference is derivative spectrophotometry. Derivative spectrophotometry is a well established technique for the assay of drugs in mixtures and in pharmaceutical dosage forms enhancing the resolution of overlapping bands. It can be applied for the determination of a drug in the presence of another by selecting a wavelength where contribution of one compound is almost zero while the compound to be determined has a reasonable value, so it has been used in the determination of many drugs (15-21). First derivative technique showed that both MET and STG could be determined by measuring the amplitude at 246.5 nm and 275 nm, respectively (Fig. 4). A linear correlation was obtained between the amplitude values and the corresponding concentrations for both drugs at their corresponding wavelengths. The characteristic parameters of the regression equation of the first derivative method for the determination of MET and STG are given in Table 3.

**Table 3:** Results obtained by first derivative method for the determination of sitagliptin and metformin in mixture

Item	Sitagliptin	Metformin

λ_max_ of measurements	275 nm	246.5 nm
Obedience of Beer’s law	50-300 μg ml^-1^	2-12 μg ml^-1^
Regression equation	C_μg/ml_ = 0.0031 H_275_ + 0.0046	C_mg/ml_ = 0.0359 H_246.5_ + 0.0039
Regression coefficient (r^2^)	0.9982	0.9997
LOD μg ml^-1^	14.92	0.24
LOQ μg ml^-1^	45.23	0.73
S_b_	8.25 × 10^-5^	3.2 × 10^-4^
S_a_	1.6 × 10^-2^	2.5 × 10^-3^
Confidence limit of the slope	0.0031 ± 3.69 × 10^-2^	0.0359 ± 5.8 × 10^-3^
Confidence limit of the intercept	0.0046 ± 1.9 × 10^-4^	0.0039 ± 7.4 × 10^-4^
Standard error of the estimation	0.017	0.00265
Results		
Drug in laboratory mixture	100.27 ± 0.92	99.98 ± 1.19
Drug in dosage form	99.97 ± 0.88	99.55 ± 1.64
Drug added	99.47 ± 1.62	99.92 ± 1.49

**Figure 5:**
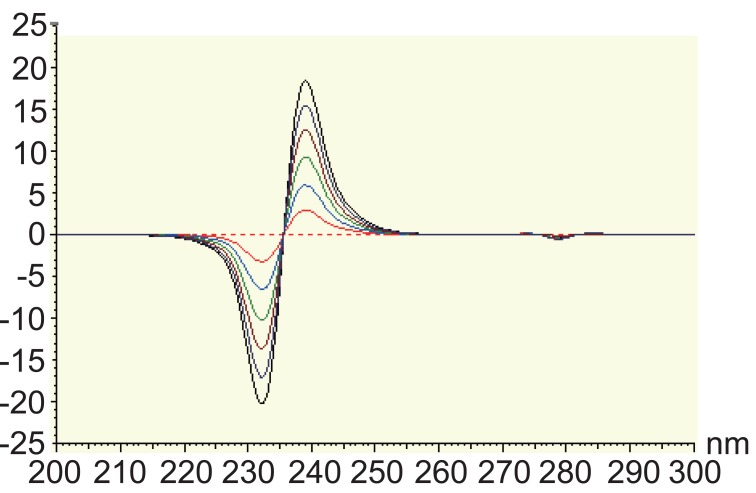
First derivative of ratio spectra of metformin hydrochloride 2-12 μg/ml using the spectrum of 50 μg/ml of STG as a divisor, methanol was used as a blank.

Another method for resolving interferences between overlapping spectra is the derivative ratio spectrophotometry method (20, 21). The absorption spectrum of the mixture is obtained and divided by the absorption spectrum of a standard solution of one of the components and then the first derivative of the ratio spectrum is obtained. MET has been determined in the concentration range of 2-12 μg/ml using the spectrum of 50 μg/ml of STG as a divisor using methanol as a blank. In order to optimize the ratio derivative method that was developed, the influence of different variables was studied. These variables included divisor concentration and smoothing factor. The careful choice of the divisor and the working wavelengths were of great importance, so ten different concentrations of STG (10, 20, 30, … and 100 μg mL^-1^) were tried as divisors. It was found that minimum noise and better selectivity were obtained upon using 50 μg mL^-1^ of STG spectrum as a divisor. Two calibration curves were constructed at 232 nm and 239 nm, representing the relationship between the amplitudes and the corresponding concentrations (Figure 5). The characteristic parameters of the regression equation of the ratio derivative method for the determination of MET are given in Table 4.

## QUANTIFICATION, ACCURACY AND PRECISION

Standard calibration curves were prepared by separately preparing series of different concentrations of the two drugs and applying the suggested procedures. The linearity of the calibration curves were validated by the high value of correlation coefficients. The analytical data of the calibration curves including standard deviations for the slope and intercept (S_b_, S_a_) are summarized in Tables 1-4. The regression equations of these calibration graphs were utilized for the determination of concentrations of the cited drugs in laboratory prepared mixtures and tablets. The reproducibility and accuracy of the suggested methods were assessed using different laboratory prepared solutions of different concentrations and determination of the concentrations in tablets. The results obtained were of good accuracy and precision. The applicability of the procedures for estimation of tablets was validated using standard addition technique as a check for accuracy (Tables 1-4).

**Table 4:** Results obtained by ratio derivative method for the determination of metformin in mixture

Item	At 239 nm	At 232 nm

λ_max_ of measurements	239 nm	232 nm
Obedience of Beer’s law	2-12 μg ml^-1^	2-12 μg ml^-1^
Regression equation	C_μg/ml_ = 1.5058 H_239_ – 0.0008	C_mg/ml_ = 1.6264 H_232_ – 0.0061
Regression coefficient (r^2^)	0.9997	0.9997
LOD μg ml^-1^	0.23	0.25
LOQ μg ml^-1^	0.70	0.77
S_b_	1.3 × 10^-2^	0.015
S_a_	9.86 × 10^-2^	0.1168
Confidence limit of the slope	1.5058 ± 0.23	1.6264 ± 0.27
Confidence limit of the intercept	-0.0008 ± 0.03	0.0061 ± 0.03
Standard error of the estimation	0.1059	0.1168
Results		
Drug in laboratory mixture	100.21 ± 0.67	99.46 ± 1.71
Drug in dosage form	100.03 ± 1.33	100.46 ± 1.44
Drug added	99.68 ± 1.05	99.71 ± 1.51

A statistical analysis of the results obtained by the proposed methods for the determination of STG and MET was carried out by “SPSS statistical package version 11”. The significant difference between groups was tested by one way ANOVA (F-test) at *p*=0.05 as shown in Tables 5-7. The test ascertained that there was no significant difference among the methods.

**Table 5:** Statistical comparison between the results of the flourometric methods and the reference methods for the determination of sitagliptin

Statistical term	Reference Method^b^	Drug in bulk	Binary mixture with MET	Binary mixture with STG alkaline degradation product^c^	Ternary mixture with MET and STG alkaline degradation product

Mean	100.5	99.83	99.93	99.81	99.94
S.D. ±	1.39	0.83	0.7	0.6	1.09
S.E. ±	0.62	0.37	0.31	0.27	0.49
% RSD	1.38	0.83	0.7	0.6	1.09
n	5	5	5	5	5
V	1.93	0.69	0.49	0.36	1.19
t (a2.306)		0.93	0.82	1.02	0.71
F (a6.39)		2.80	3.94	5.36	1.62

a Figures in parentheses are the theoretical t and F values at (*p*=0.05);

b Reference method: aliquots of standard solutions in distilled water containing 2-10 μg/ml STG were measured at 220 nm using water as a blank (1);

c STG degradation product with different ratios (10% to 30%) w/w.

**Table 6:** Statistical comparison between the results of the spectrophotometric methods and the reference method for the determination of sitagliptin

Statistical term	Reference Method^b^	Drug in bulk	Binary mixture MET	Ternary mixture with MET and STG alkaline degradation product^c^

Mean	100.5	100.13	100.27	100.47
S.D. ±	1.39	0.67	0.92	1.09
S.E. ±	0.62	0.30	0.41	0.49
% RSD	1.38	0.67	0.92	1.08
n	5	5	5	5
V	1.93	0.45	0.85	1.19
t (a2.306)		0.54	0.31	0.04
F (a6.39)		4.29	2.27	1.62

a Figures in parentheses are the theoretical t and F values at (*p*=0.05);

b Reference method: aliquots of standard solutions in distilled water containing 2–10 μg/ml STG were measured at 220 nm using water as a blank (1);

c STG degradation product with different ratios (10% to 30%) w/w.

**Table 7:** Statistical comparison between the results of the spectrophotometric methods and the refernce methods for the determination of MET in binary mixture with sitagliptin

Statistical term	Reference Method^b^	First derivative	Ratio derivative at 232 nm	Ratio derivative at 239 nm

Mean	100.4	99.98	99.46	100.21
S.D. ±	0.28	1.19	1.71	0.67
S.E. ±	0.13	0.53	0.76	0.30
%RSD	0.28	1.19	1.72	0.67
n	5	5	5	5
V	0.08	1.42	2.92	0.45
t (a2.306)		0.77	1.21	0.58
F (a6.39)		0.06	0.03	0.18

a Figures in parentheses are the theoretical t and F values at (*p*=0.05);

b Reference method: aliquots of standard solutions in distilled water containing 2-12 μg/ml MET were measured at 232 nm using water as a blank (24).

## CONCLUSION

The proposed methods have the advantages of simplicity, precision, accuracy and convenience for the quantitation of binary mixture of STG and MET in dosage form as well as ternary mixture of STG with MET and STG alkaline degradation product. The flourometric method was of higher sensitivity. Hence, the proposed methods can be used for the quality control of the cited drugs and can be extended for routine analysis of the drugs in their pharmaceutical preparations.
